# The role of dexmedetomidine administered via intravenous infusion as adjunctive therapy to mitigate postoperative delirium and postoperative cognitive dysfunction in elderly patients undergoing regional anesthesia: a meta-analysis of randomized controlled trials

**DOI:** 10.1186/s12871-024-02453-5

**Published:** 2024-02-23

**Authors:** Di Wang, Xiao He, Zicen Li, He Tao, Congjie Bi

**Affiliations:** 1 Department of Anesthesiology, Dalian Municipal Central Hospital Affiliated to Dalian University of Technology, Dalian, Liaoning, China; 2 Dalian Medical University, Dalian, China

**Keywords:** Dexmedetomidine, Non-cardiac surgery and non-neurosurgery, Postoperative delirium, Postoperative cognitive dysfunction

## Abstract

**Study objective:**

This meta-analysis aimed to assess whether continuous intravenous administration of DEX during surgery can be part of the measures to prevent the onset of postoperative delirium and postoperative cognitive dysfunction in elderly individuals following regional anesthesia.

**Methods:**

We searched the databases of PubMed, Embase, the Cochrane Library and China National Knowledge Infrastructure (by June 1, 2023) for all available randomized controlled trials assessing whether intravenous application of dexmedetomidine can help with postoperative delirium and postoperative cognitive dysfunction in the elderly with regional anesthesia. Subsequently, we carried out statistical analysis and graphing using Review Manager software (RevMan version 5.4.1) and STATA software (Version 12.0).

**Main results:**

Within the scope of this meta-analysis, a total of 18 randomized controlled trials were included. Among them, 10 trials aimed to assess the incidence of postoperative delirium as the primary outcome, while the primary focus of the other 8 trials was on the incidence of postoperative cognitive dysfunction. The collective evidence from these 10 studies consistently supports a positive relationship between the intravenous administration of dexmedetomidine and a decreased risk of postoperative delirium (RR: 0.48; 95%CI: 0.37 to 0.63, *p* < 0.00001, I^2^ = 0%). The 8 literature articles and experiments evaluating postoperative cognitive dysfunction showed that continuous intravenous infusion of dexmedetomidine during the entire surgical procedure exhibited a positive preventive effect on cognitive dysfunction among the elderly population with no obvious heterogeneity (RR: 0.35; 95%CI: 0.25 to 0.49,*p* < 0.00001, I^2^ = 0%).

**Conclusion:**

Administering dexmedetomidine intravenously during surgery can potentially play a significant role in preventing postoperative delirium and postoperative cognitive dysfunction in patients older than 60 years with regional anesthesia according to this meta-analysis.

## Introduction

The aging global population has led to a surge in surgical procedures among elderly individuals. Unfortunately, postoperative delirium (POD) and postoperative cognitive dysfunction (POCD) have become prevalent complications in this demographic, placing a substantial burden on both patients and their families. POD manifests as abnormal consciousness and impaired thinking abilities following surgery, resulting in confusion, lack of concentration, and mental disorientation [1]. On the other hand, POCD refers to the post-surgical impairment of memory, learning ability, and thinking [2]. Several risk factors contribute to the development of early POCD, including advanced age, lower educational attainment, prolonged anesthesia exposure, postoperative infections, previous surgical history, and respiratory complications. Among these factors, scientific studies suggest that age is the primary contributor to long-term cognitive impairment after surgery [3]. The medical community currently lacks a universally accepted and standardized approach to evaluate POCD, unlike the widely used Confusion Assessment Method (CAM) for assessing POD [4]. The Mini-Mental State Examination (MMSE) serves as a crucial assessment tool, offering valuable insights into cognitive impairment and facilitating the monitoring of cognitive function fluctuations. With its quantitative assessment, MMSE scores exhibit a positive correlation with a patient's cognitive function, making it a significant tool in evaluating postoperative cognitive outcomes [5].

Choosing appropriate sedation is of great significance for the prevention of POD and cognitive dysfunction in the aging population especially for surgery under regional anesthesia. Dexmedetomidine (DEX), a selective α2-adrenergic receptor agonist, exhibits various pharmacological effects, including suppression of sympathetic nervous system activity, enhancement of vagal responsiveness, reduction of blood pressure and heart rate, and alleviation of myocardial oxygen demand. Additionally, DEX provides sedative, analgesic, anti-anxiety, sleep-promoting, and memory-altering properties [6, 7]. The unique pharmacological profile of DEX positions it as an ideal choice for sedation and anesthesia management in elderly patients. In comparison to other sedative drugs, DEX stands out for its capacity to protect neurological function by regulating neurotransmitter metabolism. This characteristic makes DEX a favorable option for sedation in elderly individuals undergoing surgery, especially under regional anesthesia [8].

In recent years, some research has focused on whether continuous intravenous administration of DEX during surgery can be part of the measures to prevent the onset and further deterioration of POD and cognitive decline in elderly individuals. However, these studies have produced inconsistent results and lack systematic comprehensive analysis. A randomized controlled trial (RCT) found that compared to conventional sedative drugs, intravenous infusion of DEX can reduce the risk of POD in elderly patients while preserving better cognitive function [9]. However, not all studies have confirmed the preventive effects of DEX, and some research results indicate that DEX might potentially result in a higher incidence of other adverse events.

In order to better evaluate the preventive effect of intravenous infusion of DEX on POD and cognitive dysfunction in elderly patients, we conducted this Meta-analysis. By collecting and including RCTs that satisfy the requirements for inclusion, we systematically analyzed the preventive effect of DEX to provide more reliable evidence for clinical practice. The primary aim is to comprehensively assess DEX’s preventive effects on POD and cognitive dysfunction in elderly patients and explore its safety profile and potential side effects.

## Methods

### Search strategy

We systematically searched the databases of PubMed, Embase, and The Cochrane Library for all relevant studies from inception to Jan 1, 2023. The PubMed basic search strategy is as follows: (("delirium"[MeSH Terms] OR "delirium"[All Fields] OR ("Postoperative Cognitive Complications"[MeSH Terms] OR "Postoperative Cognitive Complications"[All Fields])) AND ("dexmedetomidine"[MeSH Terms] OR "dexmedetomidine"[All Fields])). The search strategy was designed to retrieve and include only RCT, and the language for publications on PubMed included English and Chinese. The study only included human subjects, with an age restriction of 45 years and older, and registration in the PROSPERO database with the registration number CRD42023439830 was done before the conduction of the present meta-analysis.

### Study selection

In this meta-analysis, the inclusion of studies was guided by the criteria and requirements specified as follows: (1) The research had to follow the format of RCTs; (2) Publication of the research in English or Chinese in the investigated databases or registration on the relevant experimental platform, along with the provision of corresponding results, was required. (3) Inclusion criteria specified that elderly patients aged > 60 years were included; (4) The research targets are patients undergoing lower limb surgery and receiving regional anesthesia. (5) Infusion of dexmedetomidine or similar medications via intravenous route was a requirement during surgery. Studies that were excluded included reviews, observational studies, studies without POD or POCD, studies involving non-elderly patients, studies including surgery under general anesthesia, studies involving critically ill patients or sedated patients in the Intensive Care Unit (ICU), studies that administered DEX by a different route, and studies with repeated reports. Additionally, if only mean age values were provided at baseline or if the authors did not provide a detailed description of the randomization method and DEX management strategy, the article or trial had to be regretfully excluded.

### Data extraction

Data extraction was carried out based on standardized forms. In instances of uncertainty, we engaged in discussions with the corresponding author to address the issue. Based on the judgment of the corresponding author, the differences between the participants were ultimately resolved. The data types extracted and included in the table are as follows: first author, publication year and country, the number of patients included in the experimental and control groups, range of age, and Grade of ASA, Anesthetic type, surgery procedure, drugs applied in the control group, the anesthesia depth monitoring, the strategy of DEX infusion, cognitive problem and the assessment method.

### Assessment of trial quality

We use the Cochrane Risk of Bias 2.0 (RoB 2.0) to assess bias risk. Two researchers utilized this tool to conduct independent assessments of the quality of each trial, and if there were any differences in opinions, the final judgment was made by the corresponding author. The tool consists of six quality items: Random sequence generation (selection bias), Allocation concealment (selection bias), Blinding of participants and personnel (performance bias), Blinding of outcome assessment (detection bias), Incomplete outcome data (attrition bias), Selective reporting (reporting bias), and Other bias [10]. Each item is classified as having a low, unclear, or high risk of bias.

### Statistical analysis

The statistical analysis and graphing in this study were conducted using Review Manager software (RevMan version 5.4.1) and STATA software (Version 12.0). The evaluation of dichotomous data involved the calculation of risk ratios (RR) along with a 95% confidence interval (CI). According to the “Cochrane Handbook”, heterogeneity in meta-analysis is classified into three categories: low (I^2^ < 50%), moderate (50% ≤ I^2^ ≤ 75%), and high (I^2^ > 75%) [11]. We summarized the data using the fixed-effect model if I2 was less than 50%. Conversely, for moderate or high heterogeneity, we employed a random effects model to analyze the study’s conclusions. Finally, to determine the stability of the statistical analysis outcomes, we executed a sensitivity analysis and a visual inspection of the funnel plot.

## Results

### Search results

**Fig. 1:**
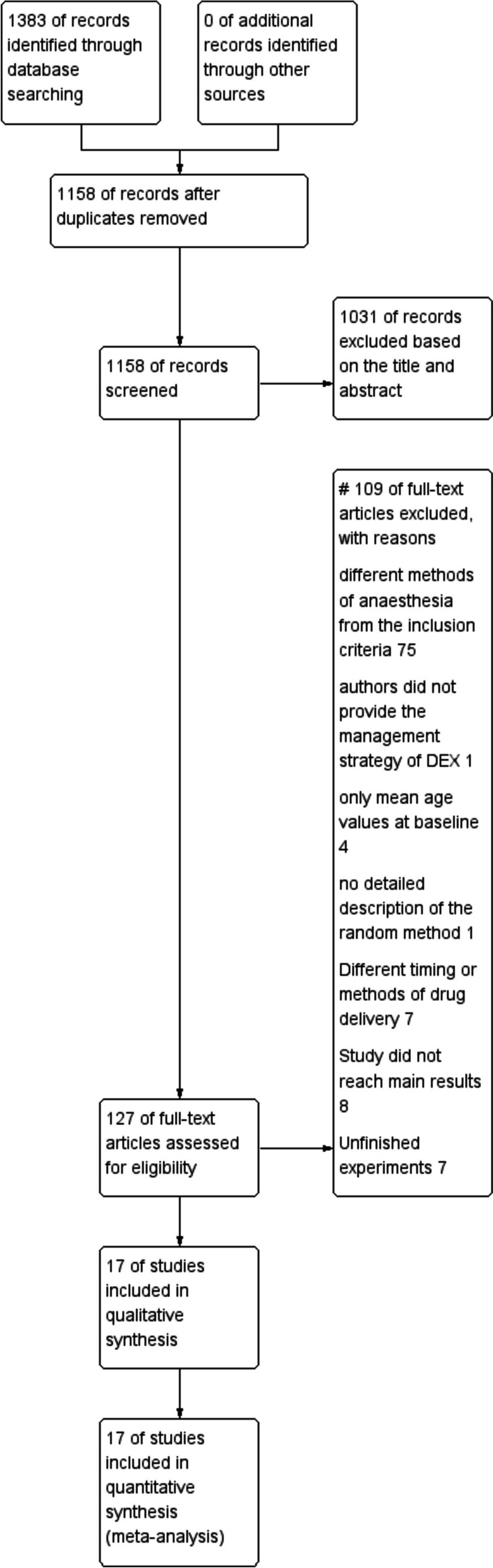
Flow diagram showing results of search and reasons for exclusion of studies

Illustrated in Fig. 1 is the comprehensive procedural journey of our literature search and database inclusion. Our meticulous search strategy across diverse databases yielded a total of 1383 articles or trials, with no additional findings from external sources. Post the elimination of duplicate entries, we were left with 1158 distinct articles for subsequent consideration. Based on the evaluation of paper headings and summaries, the two researchers collectively excluded 1031 articles and related experiments, primarily due to their low relevance to the objective of undertaking this meta-analysis. We subsequently conducted a thorough review of the remaining 127 articles and excluded 110 articles based on pre-established criteria. In total, the final meta-analysis incorporated seventeen RCTs that met the inclusion criteria.

### Characteristics of trials

The meta-analysis encompassed studies published within the timeframe of 2016 to 2022. A total of 2763 patients participated in the trials, with 1356 patients receiving intraoperative intravenous dexmedetomidine for sedation, and the control group, comprising 1407 patients, received other medications or none at all. The studies, published on various platforms in either English or Chinese, included 15 conducted in China, and one each in India and Korea. All enrolled participants were aged over 60 years. Our meta-analysis included nine studies with a total of 1955 patients, which thoroughly investigated the association between DEX and a decrease in the incidence of POD. Additionally, to investigate the potential of DEX in reducing the incidence of POCD, 808 individuals were involved as participants in the 8 studies included in the meta-analysis. Table 1 provides characteristics of the included trials.

**Table 1:** The characteristics of the included studies

**Study ID**	**Country**	**Design**	**Group**	**Anesthetic type**	Surgical procedure	Grade of ASA	Age range (years)	Control	**Anesthsia depth momotoring**	**Strategy of DEX**	**Cognitive problem**	**Assessment or define**
Dr.Deb Sanjay Nag 2020 [12]	India	2-arm RCT	DEX60 Control 60	Spinal Anesthesia	Hip surgery	I-III	60–75	NS	NR	Loading dose of DEX 1 mcg/kg over 10 min, after Spinal Anaesthesia and before start of surgery, followed by a continuous infusion ( 0.4 mcg/kg/h) until the end of surgery	POCD	MoCA scores below 26 were considered as POCD
Tianqiu Zhu 2019 [13]	China	2-arm RCT	DEX32 Control 32	combined spinal-epidural anesthesia	Hip surgery	II-III	72 +	NS	NR	followed by a continuous infusion (0.3 μg/ kg/h)	POCD	NR
Zhengxuan Guo 2017 [14]	China	3-arm RCT	DEX30 Control60	combined spinal-epidural anesthesia	hip replacement	II-III	65–92	NS/Midazolam	NR	Loading dose of DEX 0.6 μg/kg in 15 min and followed by a continuous infusion (0.2–0.5 μg/ kg)	POCD	MMSE scores of 24–27 are considered mild POCD, 19–23 are moderate POCD, and 0–18 are severe POCD
Jie Gao 2018 [15]	China	2-arm RCT	DEX47 Control47	combined spinal-epidural anesthesia	Knee Arthroplasty	II-III	60–77	NS	NR	Loading dose of DEX 1 μg/kg/h in 10 min and followed by a continuous infusion (0.5 μg/ kg/h)	POCD	A reduction of ≥ 2 points in MMSE score compared with preoperative period can be determined as POCD
Xinglong Qin 2016 [16]	China	2-arm RCT	DEX40 Control40	combined spinal-epidural anesthesia	lower extremity orthopedic surgery	II-III	65–93	NS	NR	Loading dose of DEX 0.5 μg/kg/h in 10 min and followed by a continuous infusion (0.4 μg/ kg/h)	POCD	A reduction of ≥ 2 points in MMSE score compared with preoperative period can be determined as POCD
Xiue Zhao 2018 [17]	China	2-arm RCT	DEX30 Control30	combined spinal-epidural anesthesia	lower extremity orthopedic surgery	I-III	65 +	NS	NR	Loading dose of DEX 1 μg/kg/h in 15 min and followed by a continuous infusion (0.2–0.7 μg/ kg/h)	POCD	MMSE scores ≤ 23 were considered as POCD
Yu Qi 2020 [18]	China	2-arm RCT	DEX100 Control100	epidural anaesthesia	lower extremity orthopedic surgery	I-II	65 +	NS	BIS	Loading dose of DEX 0.5 μg/kg/h in 10 min and followed by a continuous infusion (0.2 μg/ kg/h)	POCD	MMSE scores ≤ 23 were considered as POCD
Jiong Shi 2021 [19]	China	2-arm RCT	DEX66 Control66	combined spinal-epidural anesthesia	NR	I ~ III	60 +	NS	Ramsay	Loading dose of DEX 0.5 μg/kg/h in 15 min	POCD	the patient's postoperative reversible and fluctuating acute mental confusion syndrome is defined as POCD, which includes inattention, incoherent thinking, and decreased social activity ability
Xiaowei Zhang 2020 [20]	China	2-arm RCT	DEX59Control59	Spinal Anesthesia	Knee Arthroplasty	I-II	60–82	blank	NR	Loading dose of DEX 0.5 μg/kg/h in 10 min and followed by a continuous infusion (0.2 μg/ kg/h)	POD	NR
Bin Mei 2018 [21]	China	2-arm RCT	DEX148 Control 148	nerve block	Hip Arthroplasty	I–IV	65 +	propofol	BIS	Loading dose of DEX0.8–1 μg/kg in 15–20 min, after Spinal Anaesthesia and followed by a continuous infusion (0.1–0.5 μg/ kg/h)	POD	CAM
Bin Mei 2020 [22]	China	2-arm RCT	DEX183 Control 183	Spinal Anesthesia	Knee Arthroplasty	I-IV	65 +	propofol	BIS	Loading dose of DEX0.8–1 μg/kg in 15–20 min and followed by a continuous infusion (0.1–0.5 μg/ kg/h)	POD	CAM
Shin 2023 [23]	Korea	2-arm RCT	DEX366 Control 366	Spinal Anesthesia	lower extremity orthopedic surgery	I-II	65 years or older	Propofol	OAA/S	Loading dose of DEX1 μg/kg in 10 min and followed by a continuous infusion (0.1–0.5 μg/ kg/h)	POD	CAM
Feng Zhou 2022 [24]	China	4-arm RCT	DEX21 Control 63	combined spinal-epidural anesthesia	Hip surgery	I-III	65 years or older	Esketamine/NS/Esketamine + dexmedetomidine	NR	Loading dose of DEX0.6 μg/kg in 10 min and followed by a continuous infusion (0.3 μg/ kg/h)	POD	CAM
Xuan Wang 2020 [25]	China	2-arm RCT	DEX57 Control 57	combined spinal-epidural anesthesia	Hip surgery	I–IV	65 +	NS	NR	Loading dose of DEX0.5 μg/kg in 10 min and followed by a continuous infusion (0.4 μg/ kg/h)	POD	CAM
Yali Ge 2021 [26]	China	2-arm RCT	DEX52 Control52	combined spinal-epidural anesthesia	hip replacement	I ~ II	60–80	NS	NR	Loading dose of DEX 0.5 μg/kg in 15 min and followed by a continuous infusion (0.4 μg/ kg/h)	POD	CAM
Wenqing Xu 2020 [25]	China	2-arm RCT	DEX40 Control40	Spinal Anesthesia	Hip surgery	II-III	80–95	NS	NR	followed by a continuous infusion (0.2 μg/ kg/h)	POD	CAM
Haijun Weng 2022 [27]	China	2-arm RCT	DEX41 Control41	combined spinal-epidural anesthesia	orthopedic surgery	I ~ II	65 +	NS	NR	Loading dose of DEX0.4 μg/kg/h in 15 min and followed by a continuous infusion (0.2 μg/ kg/h)	POD	NR

### Risk of bias in included studies

**Fig. 2:**
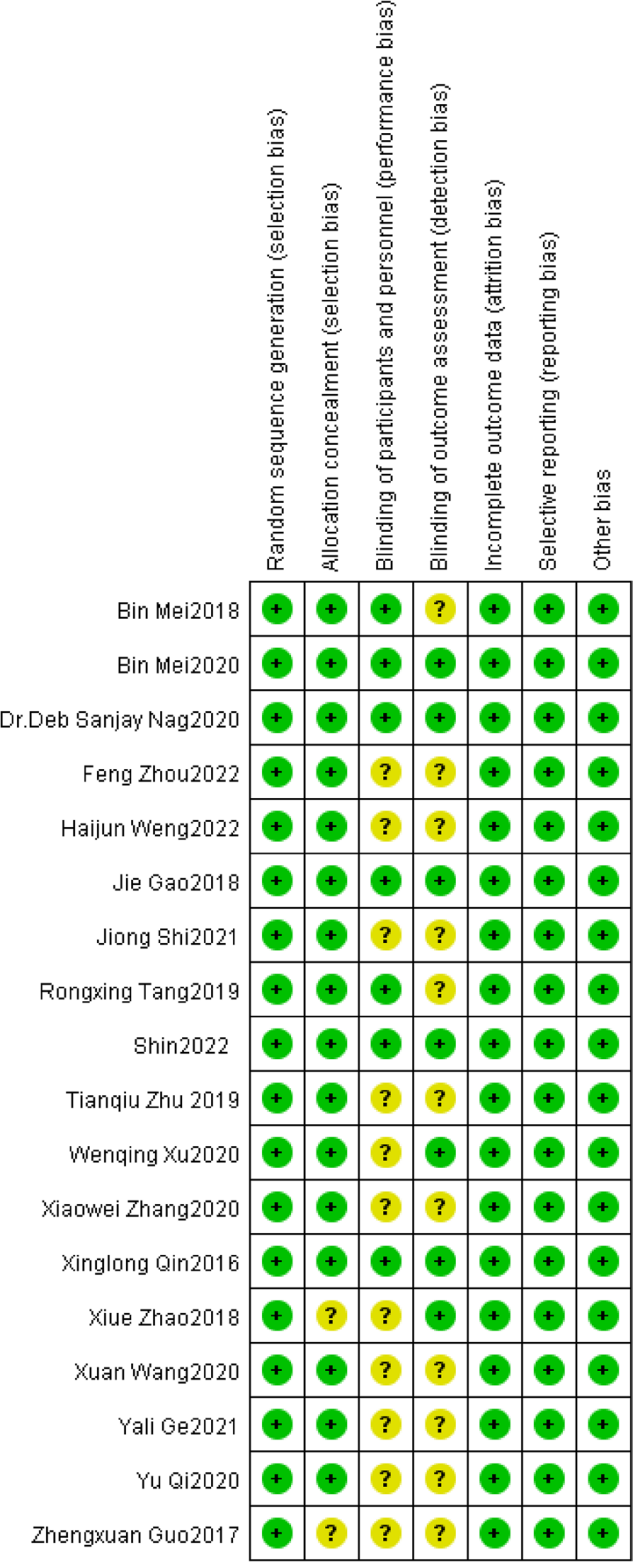
Quality assessment of each study included in the meta-analysis

**Fig. 3:**
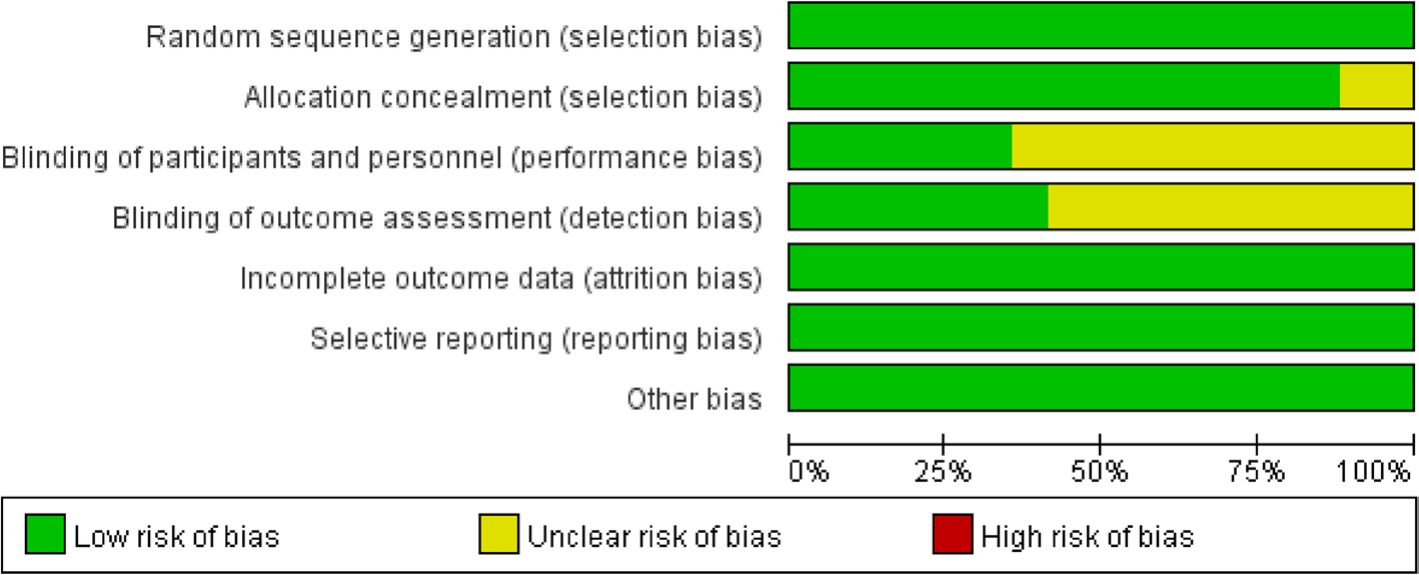
Risk of bias graph. Review authors’ judgments about each risk of bias item presented as percentages across all included studies

The primary outcome assessment was based on the RoB 2.0 tool, which was used to evaluate 17 RCTs. The risk of bias in the included RCTs was summarized and presented in Figs. 2 and 3. The analysis revealed that 12 trials had “some concerns” while 5 trials presented a “low risk of bias”.

### Meta‐analysis for the studies of cognitive problem

#### POD

**Fig. 4:**
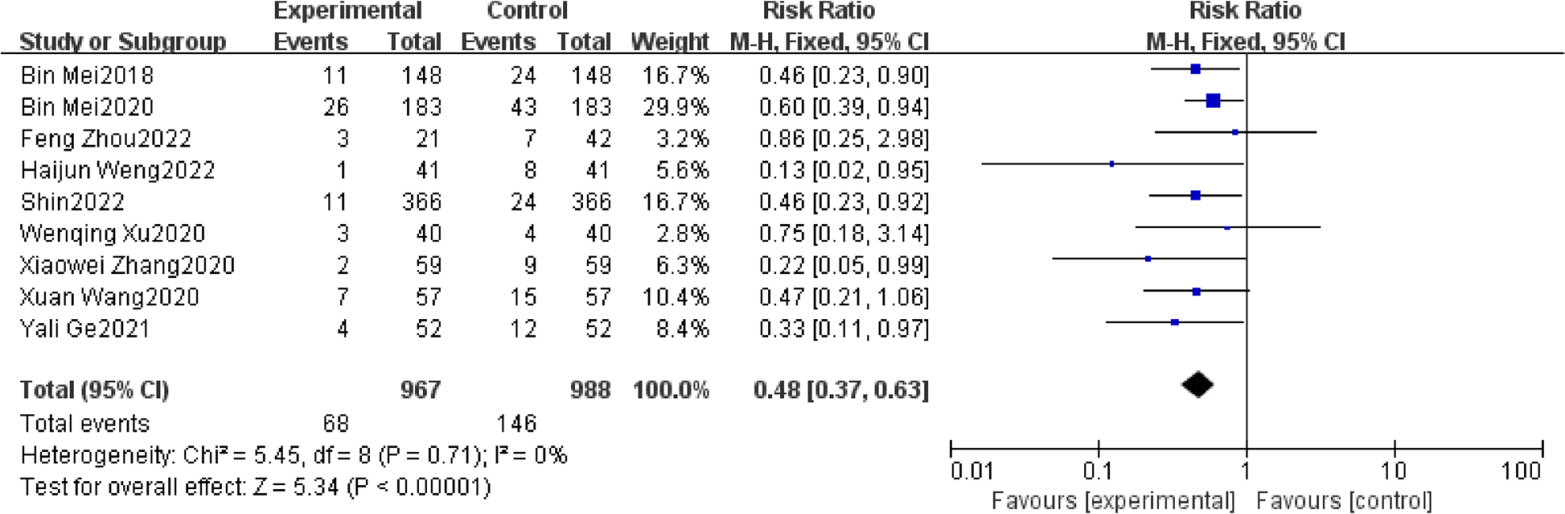
Forest plot of comparison: DEX vs Control, Outcome: incidence of POD

**Fig. 5:**
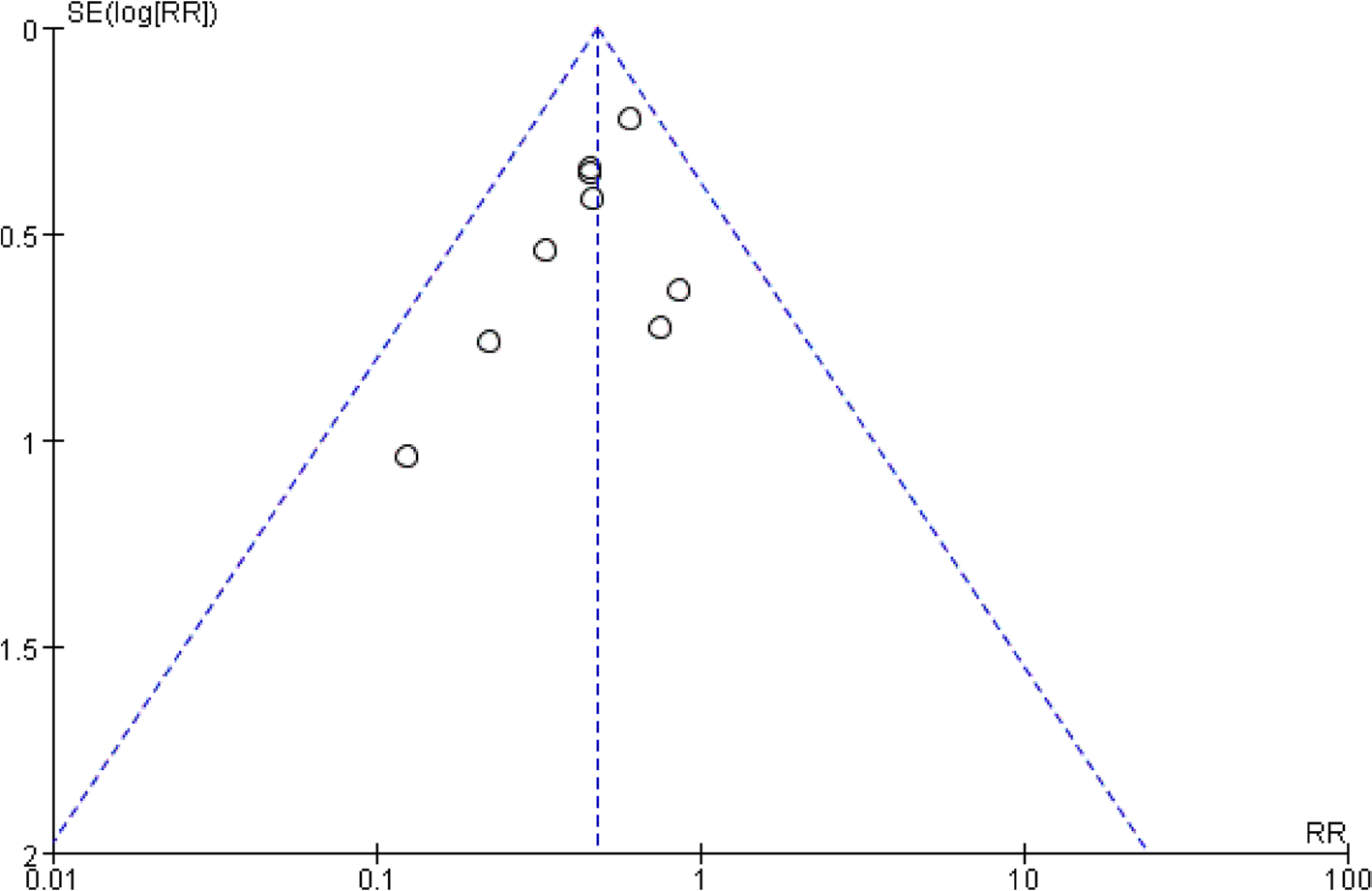
Funnel plot of the incidence of POD

**Fig. 6:**
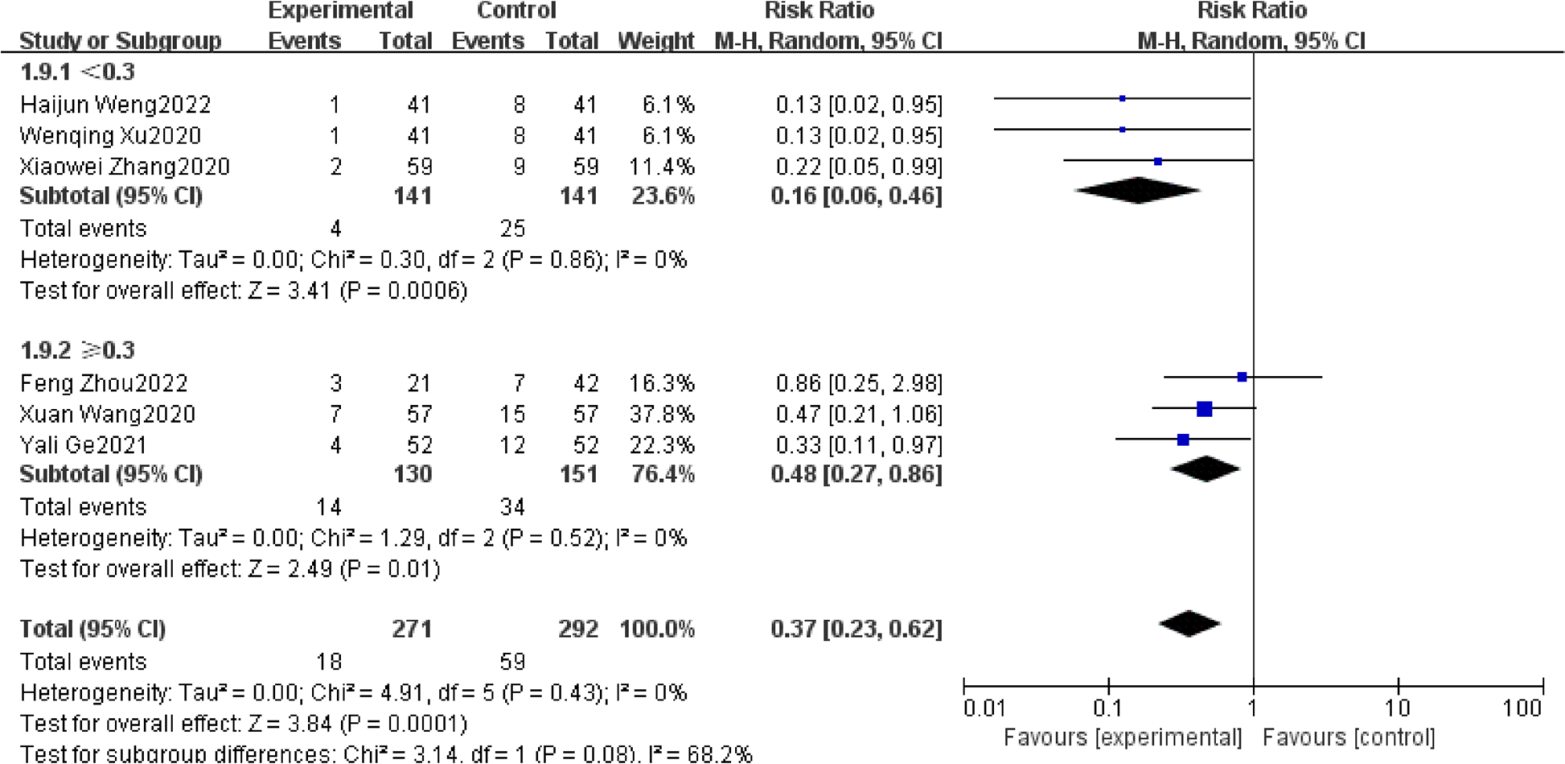
Forest plot of POD by subgroup based on the infusion rate

Meta-analysis with 1955 patients of all included studies [20–28], showed that in the case of regional anesthesia in the elderly, Evidence suggested that DEX significantly contributed to reducing the occurrence of POD (RR: 0.48; 95%CI: 0.37 to 0.63). There was insignificant heterogeneity with an I^2^ = 0%. The Z-value was also significant (*p* < 0.00001) (Fig. 4). Upon thoroughly reviewing the funnel plot through visual inspection (Fig. 5), no indications of publication bias were observed. Through a comprehensive meta-analysis involving these 9 studies, it was determined that the intraoperative administration of DEX effectively decreased the occurrence of POD in comparison to the control group, demonstrating statistical significance. Subgroup analysis comparing infusion rates after the loading dose of < 0.3 μg/ kg/h and ≥ 0.3 μg/ kg/h showed similar outcomes [RR 0.16, 95% CI (0.06 to 0.46), I^2^ = 0%, Z value (*P* = 0.0006) for infusion rate after the loading dose of < 0.3 μg/ kg/h VS RR 0.48, 95% CI (0.27 to 0.68), I^2^ = 0%, Z value (*P* = 0.01) for ≥ 0.3 μg/ kg/h] (Fig. 6.).

#### POCD

**Fig. 7:**
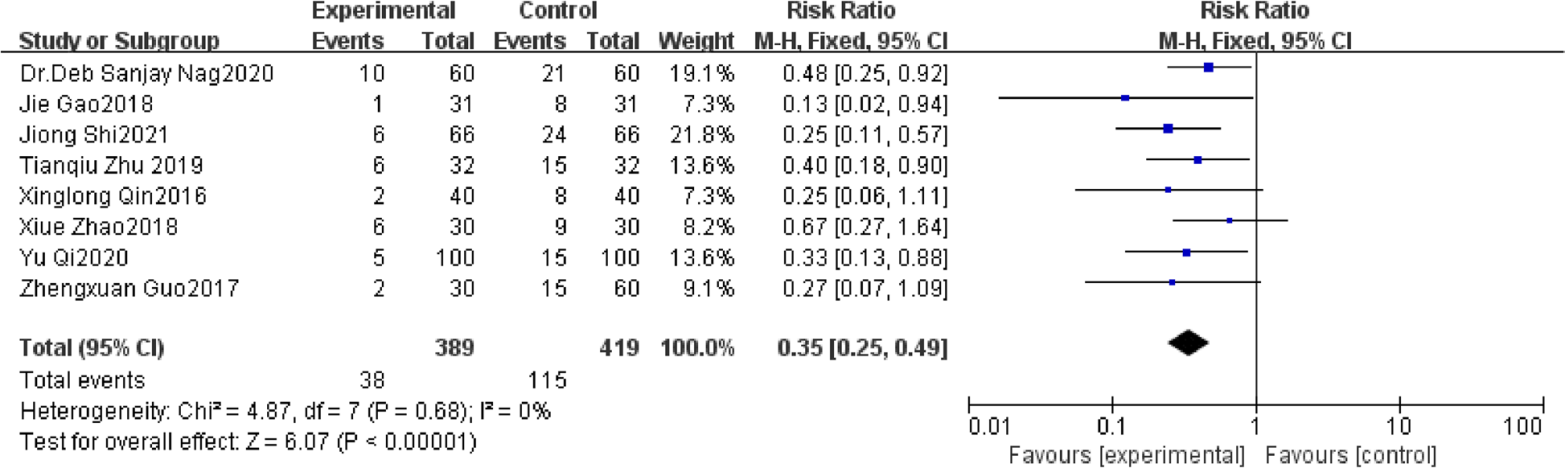
Forest plot of comparison: DEX vs Control, Outcome: incidence of POCD

**Fig. 8:**
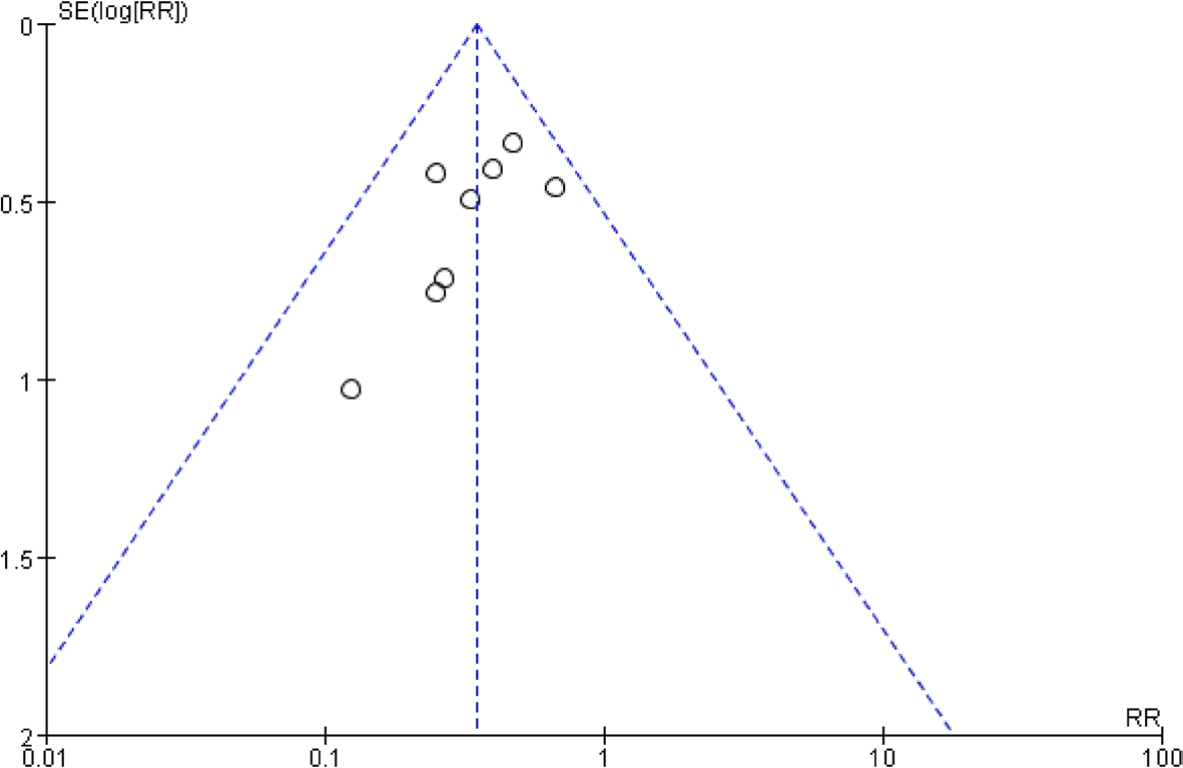
Funnel plot of the incidence of POCD

Eight studies [12–19] reported POCD, including 808 patients. We found DEX favored a reduction in POCD as reported in Fig. 7 [RR: 0.38; 95%CI: 0.27 to 0.53]. There was insignificant heterogeneity with an I^2^ of 0%. The Z-value was significant (*p* < 0.00001). The visual examination of the funnel plot (Fig. 8) did not identify any signs of publication bias. The findings from the meta-analysis, which included the examination of these 8 studies, indicated a statistically significant decrease in the prevalence of POCD while continuously delivering DEX intravenously throughout the surgery, as compared to the control group.

### Publication bias

**Fig. 9:**
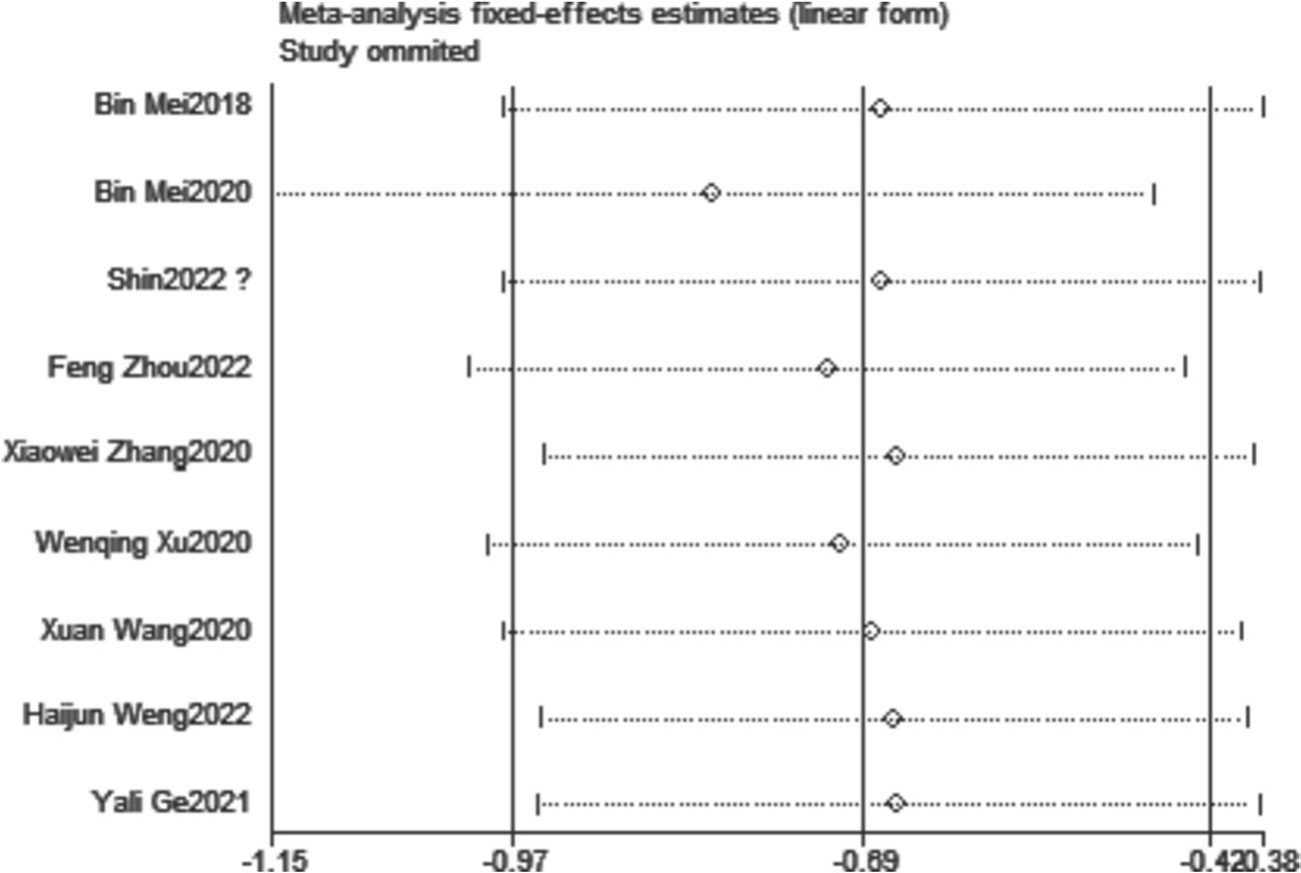
Sensitivity analysis for POD

**Fig. 10:**
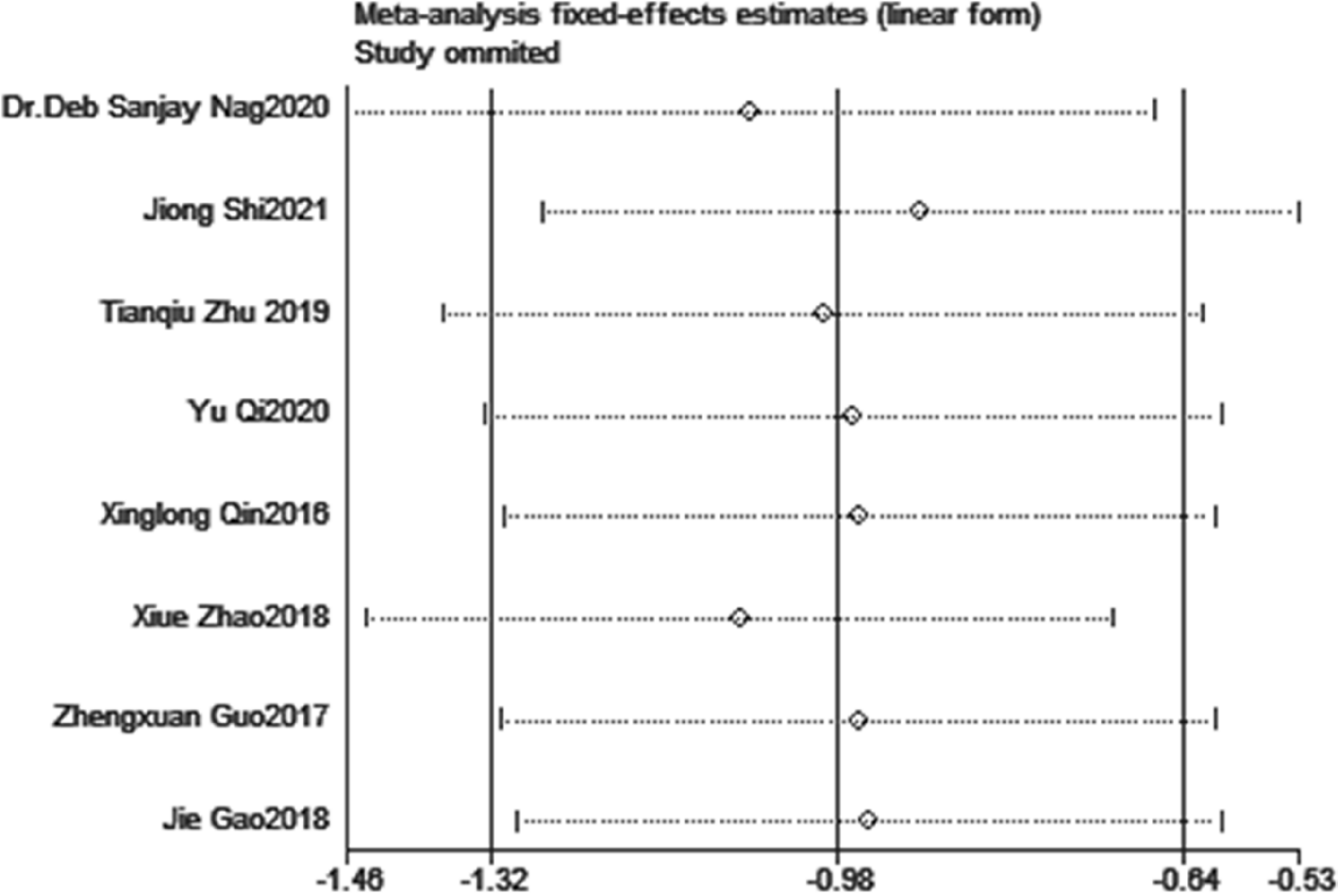
Sensitivity analysis for POCD

During the sensitivity analysis, we systematically examined the impact of excluding each study one by one from the meta-analysis. Remarkably, we observed that regardless of excluding any particular study, the overall results and conclusions of the primary outcomes remained robust and unchanged. This is effectively showcased in Figs. 9 and 10, providing visual evidence of the consistent findings throughout the sensitivity analysis. The results of Egger’s tests, with a *P* value of 0.147 for POD and 0.096 for POCD, provided no evidence of significant publication bias in the studies exploring the relationship between DEX administration and both POD and POCD.

## Discussion

This meta-analysis compared the effect of intravenous infusion of DEX with other medications or no medication control in elderly patients undergoing regional anesthesia, aiming to determine the incidence of POD or POCD. The findings demonstrated a substantial decrement in the relative risk of POD and POCD in association with intravenous infusion of DEX. Additionally, our subgroup analysis provided compelling evidence that DEX had a consistent preventive effect on POD in patients undergoing regional anesthesia, regardless of the infusion rate after the loading dose.

This meta-analysis included seventeen published articles and one completed study found in a clinical trial registry platform that provided results. All participants included in the meta-analysis were above the age of 60, ensuring a focus on the elderly population for a more targeted investigation. All studies, except for one, examined lower limb orthopedic surgeries, which limited the applicability of this meta-analysis, especially for urological and gynecological procedures where regional anesthesia was also applied to.

POD and POCD are common and life-threatening complications in the elderly. POCD can occur either after POD or independently after surgery, and it is represented by the gradual deterioration of cognitive function following a surgical procedure. POD can be observed in approximately 5% to 15% of postoperative cases. Furthermore, what is even more notable is that in specific high-risk groups, particularly among hip fracture patients, the average incidence significantly escalates, reaching a staggering 35% [29]. Defining and diagnosing POCD is challenging due to the various definitions and tests used. POD and POCD result in longer hospital stay, increased medical expenses, and reduced quality of life. These complications are associated with several risk factors, including patients of advanced age, pre-existing dementia conditions, hearing loss or visual impairment, impaired cognitive function, and metabolic/physiological disruptions [30].

The potential link between the prevention of POD and POCD may be associated with the distinct characteristics of DEX, which include an intrinsic “delirium protective effect” and may reduce the use of sedative drugs that can cause delirium, such as benzodiazepines. DEX has neuroprotective effects, which may be attributed to its potential anti-delirium effects. It does not affect γ-aminobutyric acid (GABA) receptors and does not exhibit anticholinergic activity. Additionally, research indicates that DEX aids in the fostering of physiological sleep patterns [31]. In addition, dexmedetomidine also exhibits anti-inflammatory effects by reducing key markers of inflammation such as interleukin-6 (IL-6) and interleukin-8 (IL-8), as well as tumor necrosis factor-alpha (TNF-α) [32].

In two meta-analyses that were published in 2019 [33] and 2022 [34], it was concluded that perioperative dexmedetomidine was better for postoperative neurocognitive function following surgical procedures in the elderly population, excluding cardiac surgery; although these meta-analyses only studied the non-cardiac surgery under general anesthesia. However, it is important to acknowledge that these meta-analyses focused solely on elderly patients receiving general anesthesia, overlooking the potential advantages of utilizing alternative techniques such as CSEA or nerve block. Considering patients with fragile brain function, these techniques may be more beneficial for postoperative recovery and can help prevent postoperative delirium and cognitive dysfunction. Therefore, a thorough meta-analysis was undertaken to analyze in detail the influence of DEX on the occurrence of both POD and POCD in elderly patients undergoing regional anesthesia.

Our meta-analysis affirms previous research by demonstrating that the administration of DEX to patients undergoing regional anesthesia significantly improves postoperative cognitive function. Moreover, our findings provide novel insights by showcasing the effectiveness of a continuous intraoperative infusion of DEX in preventing both POD and POCD. Subgroup analysis shows consistent preventive effects of DEX on POD, regardless of whether the maintenance dose of DEX is ≥ 0.3 μg/kg/h or < 0.3 μg/kg/h. These findings indicate that DEX has the potential to be a valuable intervention in perioperative care for patients with fragile brain function. Further investigation in future studies is warranted to explore its potential benefits and optimize its use in this population.

This study, though valuable in certain aspects, has inherent limitations and shortcomings. Firstly, a significant aspect is that some of the studies included in this meta-analysis had relatively lower research quality. Secondly, a notable limitation is the lack of reporting on intraoperative and postoperative adverse reactions in the included studies. The absence of such data prevents a comprehensive assessment of the risks associated with the administration of DEX during regional anesthesia. Furthermore, it should be noted that all the studies included in this meta-analysis were conducted solely in Asian countries. While these findings provide valuable insights, it is essential to consider the potential influence of geographical and ethnic factors on the outcomes. Therefore, additional research from diverse populations and different regions is necessary to validate the consistency and generalizability of the results. Given these limitations, further well-designed studies with higher research quality are warranted to improve upon the research limitations and provide more robust evidence regarding the use of DEX in perioperative care for patients with fragile brain function.

Although there are limitations to our study, it stands as the inaugural meta-analysis exploring the preventive efficacy of dexmedetomidine (DEX) in mitigating the incidence of postoperative delirium (POD) and postoperative cognitive dysfunction (POCD) within the elderly surgical population undergoing regional anesthesia. Our analysis involved a meticulous systematic evaluation, adhering rigorously to meta-analysis methodologies, wherein the relevant literature was thoroughly examined and summarized. Furthermore, our study only included randomized clinical trials, which strengthened the creditability of outcomes. We hope that our study can provide some references and help for future clinical practice and related studies.

## Conclusion

In summary, dexmedetomidine reduces the incidence of POD and POCD in the elderly population undergoing surgery of the lower extremities with regional anesthesia. Although this study provides evidence for the potential of DEX in decreasing the occurrence of POD and POCD, the lack of reporting on intraoperative and postoperative adverse reactions implies certain constraints on this meta-analysis, and for this reason, a larger sample size meta-analysis is needed to help confirm our conclusion.

## Data Availability

No datasets were generated or analysed during the current study.
